# Mechanisms and heterogeneity in the construction of network infrastructure to help rural households bridge the “digital divide”

**DOI:** 10.1038/s41598-023-46650-7

**Published:** 2023-11-07

**Authors:** Xiangtai Meng, Xinting Wang, Ubair Nisar, Shiying Sun, Xin Ding

**Affiliations:** 1 College of Economics, Shandong University of Technology, Zibo, China; 2 ANU College of Science, Shandong University, Weihai, China

**Keywords:** Environmental economics, Socioeconomic scenarios

## Abstract

Addressing the digital divide that plagues rural areas has become an important issue in narrowing the urban–rural gap and achieving common prosperity. This article examines the impact of network infrastructure on rural households’ ability to cross the digital divide by using the "broadband rural" strategy as a proxy variable for network infrastructure and combining data from the China Family Panel Studies (CFPS) with a score propensity matched difference-in-differences model (PSM-DID). The results show that network infrastructure can help farmers cross the access and use divide, but does not contribute significantly to crossing the ability divide in the current period. A triple difference model (DDD) was introduced to test the effect of network technology training on the contribution of network infrastructure to the ability gap, and the ability gap needs to be based on the use gap, so there is a delay in the response of the ability gap to policy. Further analysis reveals that network infrastructure mainly facilitates non-farm occupational groups to cross the capability divide, and facilitates middle-aged and young people to cross the digital divide, and does not have significant effects on groups involved in agricultural work and older people. In view of this, the network infrastructure should be continuously promoted, public service training on digital skills should be organized, electronic products and information services should be created exclusively for the elderly group and the group involved in agricultural production, and the ability of farmers to apply the network to their production life should be strengthened.

## Introduction

With the update and iteration of information technology, the Internet has realized the all-round penetration of people's lives. The 46th Statistical Report on the Development of China's Internet by China Internet Network Information Center (CNNIC) shows that as of June 2020, the size of China's Internet users reached 940 million, including 932 million cell phone users, and the Internet penetration rate reached 67%. However, the scale of Internet users in rural areas is 285 million, accounting for only 30.2% of the national proportion of Internet users, and there is also a large gap between digital applications and urban areas, creating a serious digital divide problem. The urban–rural digital divide not only widens the income gap between urban and rural areas^1^, but also "excludes" some enterprises and people from the fruits of the digital economy, preventing them from enjoying digital opportunities and dividends fairly, thus limiting the healthy and sustainable development of the economy and society^2^. Putting aside macro comparisons, the digital divide is also prevalent among individuals, especially in rural areas, and studies have shown that the digital divide can increase the incidence of poverty^3^ among farmers and prevent them from getting out of poverty and getting rich. Therefore, addressing the digital divide in rural areas is an inevitable requirement to narrow the urban–rural gap and achieve common prosperity. To this end, the State has launched a "broadband countryside" strategy in an attempt to accelerate the development and popularization of broadband in rural areas and to bridge the digital divide. So, will the Broadband Rural Strategy actually help farmers cross the digital divide? In view of this, the article uses microdata from the China Family Panel Studies (CFPS) from 2010 to 2018, and a quasi-natural experiment based on the "Broadband Rural" policy, to test whether the construction of network infrastructure can help rural households to cross the digital divide, and the heterogeneity of its effects on different groups.

In the 1990s, the concept of "digital divide" was introduced, and Alvin Toffler pointed out in "The Transfer of Power" that the "electronic divide" is "a divide in information and electronic technology"^4^, where the "electronic divide" is also the later digital divide. A more official definition of the "digital divide" was proposed by the National Telecommunication and Information Administration (NTIA) in its 1999 report "Left Behind in the Web: Defining the Digital Divide," which considered the digital divide to be "the gap between the information rich and the information poor.". In the twenty-first century, the issue of "digital divide" has been widely discussed around the world, and China has also held high-level seminars to discuss the nature of the digital divide and measures to address it. The digital divide" is defined as the gap between different social groups in the possession and use of modern information technology, but there is no unified definition of the digital divide in the world. The above definition of the digital divide informs the authors, in the author's view, The digital divide refers to the gap between various types of subjects in terms of their ability to master, own and control digital technologies. But the digital divide does not necessarily need to be compared; it can be seen as a naturally occurring threshold, and crossing the threshold means crossing the digital divide.

But the "digital divide" is a general concept that requires in-depth analysis in order to study the digital divide. Therefore, research has broken down the digital divide into different levels. For example, the digital divide at the technology base level is often referred to as the "access divide" or the "first digital divide," while the digital divide at the technology application level is referred to as the "usage divide "or the second digital divide"^5^, these two divisions have become recognized as a more authoritative classification, and it is usually believed that crossing these two divisions is to solve the problem of the digital divide of individual existence. But with the improvement of digital level, the digital divide from two levels of differentiation began to seem not scientific enough, on the basis of the access divide and the use of the divide, experts and scholars have proposed a deeper level of digital divide such as ability divide^6^, learning divide^7^. Whether it is the ability divide or the learning divide, both put forward higher requirements for crossing the digital divide, requiring individuals to improve themselves through digital applications and integrate digital into all aspects of production and life. But in contrast, the capability divide encompasses the learning divide, on this basis, the digital divide is categorized into an access divide, a usage divide and an ability divide according to farmers' access to and ability to apply the Internet. These three classes can be well categorized into three groups of farmers, that is, those who have access to the network, those who can use the network and those who can apply the network to their productive lives and self-improvement, and there is a gradual increase in the digital capacity of the three groups. Although there are abundant studies on the division of digital divide levels and the measurement of the digital gap between regions, there is still a gap in exploring how to choose indicators to measure and measure each divide from the perspective of micro-individuals.

In previous studies, the main ideas in narrowing the rural digital divide include bridging it by moderately increasing the construction of network infrastructure and e-government in the central and western regions, lowering the cost of purchasing digital technology products and services in rural areas, and vigorously developing digital inclusive finance^8^. In particular, network infrastructure development is the basis for the above responses. With regards this, China started to implement the "Broadband China" strategy in August 2013, elevating the network infrastructure construction to a national strategy, and under the planning of the general objective of the "Broadband China" strategy, in June 2014 The National Development and Reform Commission, the Ministry of Finance and the Ministry of Industry and Information Technology jointly issued a document to organize and implement the first phase pilot project of "broadband countryside". Since the implementation of the strategy, China's Internet construction has made great progress in terms of user penetration rate, Internet tariff level and average downlink rate^9^. However, the issue of whether the broadband rural strategy has helped farmers cross the digital divide by driving network infrastructure development since its implementation in 2014 has not yet been validated.

In summary, the marginal contributions of the article may be: (1) In terms of research perspective, the digital divide is subdivided into three levels from a micro perspective: access divide, usage divide and capability divide, and measurement standards are formulated to provide new ideas for researching digital divide issues. (2) In terms of research content, in the context of the "broadband countryside" strategy and based on the assessment of the effects of the "broadband countryside" strategy, the study of the impact of digital infrastructure on the ability of farmers to cross different types of digital divides as well as the mediating role of network skills training enriches the study of farmers' cross-domain digital divides. (3) In terms of practical application, the age heterogeneity of the impact of the "Broadband Rural" strategy is examined in terms of occupational type and population aging, which will help the government and leaders to formulate corresponding countermeasure proposals for different groups to cross the digital divide.

## Theoretical analysis and research hypothesis

The impact of network infrastructure on farm households across the digital divide. Regarding network infrastructure, the existing literature affirms its positive role in promoting regional economic development^10^ and industrial structure upgrading^11^, but there is little literature on the impact of network infrastructure on crossing the digital divide. On the contrary, the development of network infrastructure has widened the digital divide between urban and rural areas and between regions has become a focus of attention^12, 13^. In comparison, it is a fact that urban areas and developed eastern regions take advantage of their economic strength and human capital to develop network infrastructure significantly faster, and the gap with lagging regions gradually widens. However, if comparisons are put aside, it is well established that network infrastructure can facilitate the crossing of the digital divide for farmers^14^. However, regarding the specific impact of cyberinfrastructure on the three tiers, the authors summarize the following three aspects. First, network infrastructure can bring about the improvement of broadband user scale, broadband penetration level, broadband network capacity and broadband information application^15^, which plays a key role in accessing the Internet and improving network quality for farmers and helps them cross the access divide. Second, the development of network infrastructure is the basis for the application of digital products and technologies, which play a positive role in enhancing the digital capacity of farmers to cross the access divide or a deeper digital divide^16^.Third, in terms of the "broadband countryside" strategy, broadband infrastructure construction is the main task and starting point, and the ultimate purpose is to solve the problem of digital divide in rural areas, which also affirms the positive role of network infrastructure construction for farmers to cross the digital divide from the national level, and the achievements of the later strategy also reaffirm this The achievements of the later strategy also reaffirm this view. However, the capacity divide is no longer limited to the development and use of digital technologies, as opposed to the access and use divide^17^, and it is more difficult to cross the capacity divide with the facilitation of network infrastructure development alone.

Hypothesis 1a: Network infrastructure development can help farmers cross the access divide.

Hypothesis 1b: Network infrastructure development can help farmers cross the usage divide.

Hypothesis 1c: Network infrastructure development does not help farmers cross the capacity divide.

The moderating effect of skills training on crossing the capability divide. Crossing the capability divide puts higher demands on the level of human capital of farmers, and related scholars define the capability divide as differences in access to, use of and creation of digital resources by different economic agents due to differences in digital literacy, and use civic literacy as a measure of the difference in the capability divide between regions^18^. From the definition, it is easy to see that improving citizens' digital literacy is the key to help rural households cross the capability divide. However, the residents in rural areas are generally less literate, and it is difficult to achieve digital literacy on their own, while the development of relevant network technology skills training can not only improve farmers' mastery of digital applications, but also contribute to crossing the capability divide by improving citizens' digital literacy. In addition, the argument that digital skills training helps farmers to cross the digital divide has been confirmed^19^.

Hypothesis 2: Web-related technical skills training can help farmers cross the capacity gap.

## Background of "broadband village"

The low level of Internet development is particularly prominent in China's rural areas, which has seriously affected China's economic and social development, in order to enhance the level of Internet development in rural areas, implement the "12th Five-Year" National Strategic Emerging Industry Development Plan and the "Notice on the Issuance of the "Broadband China In order to enhance the level of Internet development in rural areas, implement the "12th Five-Year Plan" and the "Notice on the Issuance of "Broadband China" Strategy and Implementation Plan", accelerate the development and popularization of broadband in rural areas, in 2014, the three ministries jointly organized the implementation of the "Broadband Village" pilot project. The first batch of pilot projects include Inner Mongolia Autonomous Region, Sichuan Province, Guizhou Province, Yunnan Province, Shaanxi Province and Gansu Province.In July 2014, the three ministries selected Sichuan as the first to start the implementation of the pilot project from the planning submitted by six provincial administrative units.

The specific objectives of the pilot project are: combined with the "Broadband China" strategy implementation schedule, the pilot provinces and autonomous regions to choose at least 20 counties (districts, banners) to continue to promote Internet penetration in rural areas. It is required that by 2015, more than 95% of administrative villages will be connected to optical fiber cables, rural broadband access capacity will reach 4 M and above, and rural household broadband penetration rate will reach 30%. Pilot counties (districts, banners) network infrastructure requirements in rural areas to reach 100% of the proportion of fiber optic cable, the proportion of administrative villages to 4 M and above rate broadband is less than 50%. In the specific implementation process, the "Broadband Countryside" pilot project not only ensures fiber optic access in rural areas, focusing on improving rural broadband coverage, but also focuses on the improvement of data transmission rate, that is, increasing the speed of the network. At the same time, the project facilitates rural residents to access the Internet through low-cost cell phones by vigorously developing wireless broadband and 3G, 4G, LTE and other technologies. The three ministries will carry out inspection and evaluation work from time to time to ensure that the policy achieves the expected results.

The results of the pilot project include: first, promoting the application of information technology deep into the rural grassroots government on the basis of e-government extranet; second, promoting the application of agricultural technology in rural areas to meet the information needs of farmers, forestry and livestock technical content management; third, creating a modern agricultural distribution network through e-commerce and establishing a modern agricultural distribution network through e-commerce to solve the current agricultural products production and marketing docking Third, through e-commerce, we will create a modern agricultural distribution network and establish a modern agricultural distribution network through e-commerce to solve the difficulties of the current agricultural products production and marketing. At the same time, "broadband countryside" can also enable distance education to reach rural areas, more convenient medical insurance and rural financial network can cover almost all areas. In short, the implementation of the "broadband countryside" strategy lays the foundation for the gap between urban and rural information infrastructure, bridging the urban–rural digital divide, equalizing public services, implementing local economic development, and implementing policies to benefit the people.

## Data sources, variable selection and model selection

### Data sources

The article uses data from the 2010–2018 China Family Panel Studies (CFPS), which is a survey organized and compiled and released by the China Social Science Survey Center of Peking University, which reflects the social, economic, and educational changes in China by tracking data at three levels: individual, family, and community. existing CFPS There are seven issues of public data, and due to the lack of relevant panel data in 2012 and 2020, five issues of data in 2010, 2011, 2014, 2016, and 2018 are selected as the research samples. The data are processed as follows: 1. Household member questionnaire, household economic questionnaire, and individual self-response questionnaire are combined horizontally. 2. 5 periods of data are combined longitudinally, and rural households are screened out as samples using individual households in the household economic questionnaire. 3. Central and western regions are screened out as research samples, and experimental and control groups are divided according to the policy implementation pilot, and relevant variables are retained. 4. Relevant missing values and outliers of the variables. The final research sample of 18,098 rural households was obtained.

### Variable selection

(i) *Explanatory variables* Digital divide. Experts and scholars have not yet reached a unified conclusion on the specific measurement indicators for measuring the digital divide. The article subdivides the digital divide into the access divide, the access gap refers to the fact that farmers have access to the Internet, but there is no requirement for them to be able to use it proficiently; the use gap refers to the fact that farmers have a fairly high level of proficiency in Internet application; and the competence gap refers to the fact that farmers are able to combine the use of the Internet with their work, study, etc., so as to enhance their self-worth in a comprehensive manner.

The access divide can be measured intuitively by whether or not one uses the Internet. With the deepening of digitization and the innovation of new digital applications, it is difficult to measure the access gap and the ability gap in the actual measurement process. Integrating the reasonableness of indicators and the availability of questionnaire data, the article uses online entertainment, online social networking, online shopping and online information access as the criteria to measure whether the use divide is crossed, the use of the Internet for entertainment, shopping, etc. can be a good indication that farmers have some ability to use the Internet. Compared with the ability divide, the threshold of the use divide is relatively low, and meeting three of them or more is defined as crossing the use divide; e-learning, online work and online business activities are taken as the indicators of the ability divide, it is not only a recognition of digital competence that can play an important role in the personal development of farmers. Any one of the three indicators of the capability divide can better reflect the individual's ability to apply digital capabilities, so meeting one of them is defined as farmers crossing the capability divide.

The specific indicator system is constructed as shown in Table 1.

(ii) *Explanatory variables* For network infrastructure construction, the article generates dummy variables with the implementation time and area of the "broadband village" pilot, i.e., if the area is established as a pilot in the year of the pilot implementation (2014), the value of 1 is assigned to the year and beyond, otherwise, the value of 0 is assigned as a proxy variable for network infrastructure.
(iii) *Control variables* In order to prevent the influence factors of other variables from biasing the experiment, the article draws on the research experience of Jin Chunzhi and other scholars on the digital divide problem^20^, and selects individual gender, age, years of education, marital status, engagement in agricultural production, per capita net annual household income, and the importance of information as the control variables of the study. In particular, it should be noted that household access to broadband infrastructure requires initial investment in start-up capital and regular payment of usage fees, and individual farmers with more economic power tend to have a stronger willingness to invest capital, which leads to an innate capital advantage for higher income groups. While farmers with relatively low economic level are constrained by their economic strength, which leads to reluctance to invest or insufficient investment in the early stage, making the development goals of ICT obstructed and delayed, missing development opportunities and thus expanding their own digital divide; the importance of information can enhance farmers' recognition of new things by influencing individuals' understanding of policies, thus indirectly influencing farmers' acceptance of the Internet. Prevent farmers from missing the opportunity to understand and use broadband infrastructure because of information obstruction, and help farmers cross the digital divide. The specific indicator descriptive statistics are shown in Table 2.

**Table 1:** Construction of digital divide indicators.

Dimensionality	Indicators	Indicator Description	Crossing the threshold
Access divide	Network access	Whether the farm household has access to the Internet	A value of 1 is assigned if the condition is met and 0 if not
Use of the divide	Internet Entertainment	Internet access for entertainment activities such as listening to music and watching movies	A value of 1 is assigned if three or more of these conditions are met; otherwise, a value of 0 is assigned
	Online Social	Go online for social activities such as chatting and emotional communication	
	Online Shopping	Using the internet for shopping	
	Access to information	Access to information on current affairs and politics via the internet	
Competence divide	E-learning	Study via the internet (including non-academic education)	1 if one or more of the following is met, 0 if the other is not
	Network work	Working through the web	
	Commercial activities	Business activities such as e-commerce via the internet

**Table 2:** Descriptive statistics.

Variables	Average value	Standard deviation	Minimum value	Maximum value	Sample size
Access divide	0.249	0.433	0	1	18,098
Internet entertainment	0.221	0.415	0	1	18,098
Online social	0.227	0.419	0	1	18,098
Online shopping	0.122	0.327	0	1	18,098
Network information	0.187	0.389	0	1	18,098
Use of the divide	0.114	0.318	0	1	18,098
E-learning	0.155	0.362	0	1	18,098
Network work	0.088	0.283	0	1	18,098
Commercial activities	0.069	0.206	0	1	18,098
Competence divide	0.813	1.540	0	5	18,098
Is it a pilot	0.505	0500	0	1	18,098
Gender	0.532	0.516	0	1	18,098
Age	46.584	17.169	16	95	18,098
Years of education	5.762	4.606	0	19	18,098
Marital status	0.662	0.473	0	1	18,098
Are you engaged in agricultural production	0.803	0.398	0	1	18,098
Net annual household income per capita	0.987	1.825	0	16.5	18,098
Importance attached to information	0.149	0.356	0	1	18,098

### Model selection

The article uses the exogenous shock variable "Broadband Village" pilot as a proxy variable for network infrastructure, and uses a difference-in-differences model (DID) model to test the causal effect of network infrastructure on farmers' crossing the digital divide. A propensity score matching (PSM) approach is further used to bring the data closer to the randomized experiment through nearest neighbor matching (1:2), the nearest neighbor matching method matches study subjects by treatment group, and all individuals can be successfully matched to ensure that the full sample is retained. The pilot provinces of Inner Mongolia Autonomous Region, Sichuan Province, Guizhou Province, Yunnan Province, Shaanxi Province, and Gansu Province were selected as the experimental groups for the study. Since the pilot provincial administrative units all belong to the central and western regions, and the central and western regions of China are homogeneous in terms of economic development level, infrastructure construction, and industrial structure, other central and western provincial administrative units of China are selected as the experimental control group. Considering that the pilot areas were first carried out in July 2014 (Sichuan Province), which is located between 2014 and 2016, but considering that it is closer to 2014, and the acceptance of the "broadband countryside" policy should be completed in 2016 according to the policy requirements, therefore, 2014 is taken as the current period of policy implementation. The estimation equation is constructed as follows.

1 $${Y}_{ipt}={\alpha }_{0}+{\alpha }_{1}{D}_{p(i)}\times {T}_{t}+{\alpha }_{2}{C}_{ipt}+{\beta }_{p}+{\gamma }_{t}+{\delta }_{ipt}$$

In Eq. (1), ${\mathrm{Y}}_{\mathrm{ipt}}$ indicates whether individual i in province p crosses the digital divide at time t.${D}_{p(i)}$ indicates whether individual i's province is in the experimental group of the "broadband rural" policy. ${T}_{t}$ indicates whether the factual "broadband rural" pilot work starts at time t. ${C}_{ipt}$ is a control variable controlling for its effect on the explanatory variables and DID, ${\beta }_{p}$ is a region fixed effect, ${\gamma }_{t}$ is a time fixed effect, and ${\delta }_{ipt}$ is a random disturbance term.

## Results and analysis

### Analysis of the impact of network infrastructure on farm households across the digital divide

Column (1) in Table 3 reports the regression results of network infrastructure on farmers' crossing of the access divide, and it can be found that network infrastructure significantly contributes to farmers' crossing of the access divide at the 1% significant level. Column (2) reports the results of the regression of network infrastructure on farmers' crossing the usage divide, and it can be found that network infrastructure facilitates farmers' crossing the usage divide at 1% significant level. Column (3) reports the regression results of network infrastructure on farmers' crossing the ability divide, and it can be found that the effect of network infrastructure construction on farmers' crossing the ability divide is not significant. In summary, network infrastructure construction can help farmers cross the access divide and the use divide, but the contribution to farmers crossing the ability divide is not significant. The main reason is that the main goal of digital village construction is to strengthen the rural digital infrastructure, and the popularization of the network will indeed enhance farmers' access to and simple use of the Internet, but limited by the reality of the backwardness of the human capital level of farmers, it is difficult to use the Internet in production and learning before receiving professional guidance and training.

**Table 3:** Baseline regression results.

Variables	Access divide	Use of the divide	Competence divide
	(1)	(2)	(3)
Is it a pilot $\times$ Whether to start implementing the policy	1.175*** (0.069)	0.528*** (0.057)	0.260 (0.022)
Gender	− 0.012 (0.052)	− 0.048 (0.046)	− 0.038 (0.039)
Age	− 0.098*** (0.002)	− 0.047*** (0.001)	− 0.070*** (0.002)
Years of education	0.250*** (0.007)	0.183*** (0.006)	0.224*** (0.006)
Marital status	0.054 (0.057)	− 0.213*** (0.048)	− 0.201*** (0.052)
Agricultural production	− 0.654*** (0.068)	− 0.254*** (0.057)	− 0.335*** (0.063)
Income	0.189*** (0.038)	0.142*** (0.022)	0.150*** (0.026)
Level of information importance	1.268*** (0.095)	0.924*** (0.087)	1.099*** (0.092)
Time fixed effects	Yes	Yes	Yes
Regional fixed effects	Yes	Yes	Yes
Observations	18,098	18,098	18,098
${\mathrm{R}}^{2}$	0.381	0.248	0.219

*, ** and *** indicate significant at the 10%, 5% and 1% levels respectively, with standard errors in brackets.

From the control variables, years of education, income, and information importance all significantly promote farmers to cross the digital divide at the 1% level; age and engagement in agricultural production significantly inhibit farmers to cross the digital divide at 1%; gender has no significant effect on the digital divide; marital status significantly promotes farmers to cross the use divide and ability divide at the 1% level, and has no significant effect on farmers to cross the access divide. Possible reasons are: the higher the education level and younger the age, the stronger the ability to accept new things and the greater the demand for network; network infrastructure requires certain financial investment, and households with higher income are more likely to accept the expenditure related to network services; the importance of information can promote farm households' understanding of policies related to network infrastructure and more likely to respond to related policies; compared with agricultural production, non-farm employment especially some related jobs that require the use of network, have a greater demand for network skills; marital status on the one hand is similar to age, married farmers are relatively older and therefore less able to accept new things than young people. On the other hand unmarried people may be due to disadvantages such as family economic situation and human capital, so there is uncertainty about marital status for farmers to cross the digital divide.

### Robustness test

*Discussion of endogeneity* On the issue of endogeneity, the main thing to explore is whether the selection of the pilot is random. First, the "broadband countryside" belongs to the national policy, and the first pilot provinces were selected randomly according to the requirements, and Sichuan and Yunnan were selected from the first batch of pilots on the basis of merit to take the lead in starting the implementation. Second, since the three ministries issued a pilot notice in June 2014, to July, Sichuan was selected as the first to start implementation, the response period is only one month, it is difficult for each region to respond to the policy in advance. Third, the problem of digital divide among farmers is prevalent in the central and western regions, all of which are more prominent, and the government's formulation of relevant policies will not be affected by special care for certain regions. Based on the above three points, the policy can be approximated as a quasi-natural experiment, but still needs relevant data support. Therefore, the article subsequently adopts the methods of parallel trend test and counterfactual test for further verification.

It is noteworthy that although the pilot regions have carried out the pilot work one after another, there is a sequential problem of time, in which Sichuan and Yunnan were the first to carry out and other provinces followed, and the "broadband countryside" policy also started the bidding work in other provinces one after another after 2015, which may lead to the problem of unclear grouping of the experimental and control groups. This may lead to the problem of unclear grouping of experimental and control groups. In view of this, the article draws on Wang Jiancheng's approach and uses Sichuan and Yunnan provinces, which were the first to implement the pilot project, as the experimental group, and Chongqing and Guizhou provinces, which are comparable in terms of economic development, industrial structure and geographical location, as the control group to further test the robustness of the experiment^21^. The results in Table 4 show that after replacing the control group with the control group, the regression results are still significantly positive at the 1% level and the model is more robust.

**Table 4:** Endogeneity test.

Variables	(1)	(2)	(3)
Is it a pilot $\times$ Whether to start implementing the policy	4.013*** (0.392)	1.302*** (0.108)	0.483*** (0.091)
Control variables	Yes	Yes	Yes
Time fixed effects	Yes	Yes	Yes
Regional fixed effects	Yes	Yes	Yes
Observations	3781	3781	3781
${\mathrm{R}}^{2}$	0.399	0.388	0.263

*, ** and *** indicate significant at the 10%, 5% and 1% levels respectively, with standard errors in brackets.

*Parallel trend test* The article's hypothesis is premised on the premise that the experimental and control groups should satisfy the parallel trend hypothesis before the implementation of the pilot exercise, and that they should be comparable before the policy shock. Before the policy implementation, whether or not it was a pilot region would have no effect on farm households crossing the digital divide, therefore, the article draws on the parallel trend test of Tian Dove et al. to construct the estimation model (2) and add the cross product terms of whether or not it was a pilot × whether or not the policy was implemented 1 period before and whether or not it was a pilot × whether or not the policy was implemented 2 periods before to the original equation. As shown in Table 5, whether a province was a pilot province before the policy implementation had no impact on farmers' ability to bridge the digital divide, while the pilot province promotes farmers crossing the digital divide at the 1% significant level after starting the implementation of the pilot. In summary, the experimental and control groups before policy implementation satisfy the parallel trend hypothesis that network infrastructure helps farmers cross the digital divide.

**Table 5:** Parallel trend test.

Variables	Access divide	Use of the divide
	(1)	(2)
Is it a pilot × Is the policy implemented	0.760*** (0.074)	0.422** (0.061)
Whether it is a pilot × whether it is a pre-policy implementation period 1	− 1.697 (0.074)	− 0.387 (0.059)
Is it a pilot × Is it the first 2 periods of policy implementation	− 0.735 (0.073)	− 0.222 (0.060)
Control variables	Yes	Yes
Time fixed effects	Yes	Yes
Regional fixed effects	Yes	Yes
Observations	18,098	18,098
${\mathrm{R}}^{2}$	0.395	0.250

*, ** and *** indicate significant at the 10%, 5% and 1% levels respectively, with standard errors in brackets.

2 $${Y}_{ipt}={\alpha }_{0}+\sum_{j=-2}^{1}{\alpha }_{j}{D}_{p(i)}\cdot {T}_{j}+{\alpha }_{1}{C}_{ipt}+{\beta }_{p}+{\gamma }_{t}+{\delta }_{ipt}$$

*Counterfactual test* There may be certain uncontrollable factors that affect the effect of policy shocks, which can have a large impact on the estimation results. To control for the effect of policy shocks due to these unobservable or random factors, the article uses a counterfactual test to verify the robustness of the model by generating counterfactual policy variables and testing whether the policy effect remains significant. Drawing on Bharadwaj et al. the sample control group is randomly assigned to the experimental group and the randomly generated experimental group is assumed to be subject to a policy shock in 2014 to test whether the model is significant^22^. The results in Table 6 show that after randomly assigning the experimental group to the control group, the effect of policy implementation on farm households crossing both digital divides is not significant. This indicates that the dummy policy variables cannot promote farmers to cross the digital divide, which again confirms the positive effect of the "broadband countryside" policy, and the model passes the robustness test and the conclusion is more realistic and reliable.

**Table 6:** Counterfactual tests.

Variables	Access divide	Use of the divide
	(1)	(2)
Is it a pilot $\times$ Whether to start implementing the policy	0.732 (0.101)	0.328 (0.066)
Control variables	Yes	Yes
Time fixed effects	Yes	Yes
Regional fixed effects	Yes	Yes
Observations	18,098	18,098
${\mathrm{R}}^{2}$	0.426	0.249

*, ** and *** indicate significant at the 10%, 5% and 1% levels respectively, with standard errors in brackets.

### Exploration of mechanisms for network infrastructure to help farm households cross the capacity divide

The solution to the digital divide that plagues rural areas is ultimately to achieve the overall goal of common prosperity by enhancing the digital level of farmers and applying it to their daily production and life, which will contribute to improving the quality of farmers and increasing their productivity and income. Therefore, the key to the problem is how to help farmers cross the capability gap. Although the cross product term in the baseline regression results is not significant for crossing the ability divide, the estimated coefficient is positive, considering that farmers need to cross a higher threshold to cross the ability divide, and the population quality in rural areas is generally low, so it is difficult to cross the high technology threshold, and participation in relevant technical skill training may help farmers to cross the ability divide. Therefore, the article further constructs a triple difference model (DDD) to test whether relevant technical skills training can improve the contribution of network infrastructure to farmers' ability to cross the ability divide. Column (1) of Table 7 examines the effect of technical skills training on farmers' ability to cross the capability gap. The results show that participation in relevant technical skills training can facilitate farmers to cross the capability gap. Column (2) introduces the triple differential cross product term, and the results show that the cross product term promotes farmers to cross the ability divide at 1% significant level after the introduction of relevant technical skills training. This is because farmers are constrained by backward education levels and have enormous difficulty in applying the Internet, while digital skills training can significantly improve farmers' ability to apply the Internet and help them cross the capability gap.

**Table 7:** Capacity divide mechanism test.

Variables	Competence divide	Competence divide	Competence divide	Competence divide
	(1)	(1)	(2)	(3)
Receiving technical skills training or not	0.434*** (0.087)			
Is it a pilot $\times$ Whether to start implementing the policy $\times$ Received technical skills training		0.324*** (0.064)		
Is it a pilot $\times$ Whether to start implementing the policy (with a one-period lag)			0.389** (0.059)	
Use of the divide				0.633*** (0.053)
Control variables	Yes	Yes	Yes	Yes
Time fixed effects	Yes	Yes	Yes	No
Regional fixed effects	Yes	Yes	Yes	No
Observations	18,098	18,098	18,098	18,098
${\mathrm{R}}^{2}$	0.169	0.128	0.318	0.139

*, ** and *** indicate significant at the 10%, 5% and 1% levels respectively, with standard errors in brackets.

Considering that relevant technical skills training generally requires a certain period of time to obtain capacity enhancement, this provides the possibility of a lag in the response to policy across the capacity divide. Therefore, the article uses a one-period lag in policy to continue to explore the impact of broadband infrastructure development on crossing the capability divide. On the other hand, crossing the usage divide may be the basis for crossing the capacity divide, and the above hypothesis is further tested by extracting data from the continuous follow-up farm household data to observe whether crossing the usage divide in the current period of a farm household has an impact on that farm household crossing the capacity divide in the next period.

Column (3) of Table 7 shows the impact of network infrastructure development on farmers crossing the ability divide after one period of policy implementation lag, and the results show that broadband infrastructure significantly contributes to farmers crossing the ability divide at the 5% level. Column (4) is the effect of farmers crossing the usage divide in the current period on the farmers crossing the ability divide in the next period after combining multiple consecutive follow-up periods horizontally, and the results show that farmers promote farmers crossing the ability divide in the next period after crossing the usage divide at a significant level of 1%, and through further statistics, it is found that the average response time from farmers crossing the usage divide to farmers crossing the ability divide is 1.28 periods. In summary, participation in relevant technical skills training can promote the impact of network infrastructure on crossing the competence divide; network infrastructure construction helps farmers to cross the competence divide with a slower response time; farmers crossing the use divide is the basis of farmers crossing the competence divide and promotes farmers crossing the competence divide.

### Further exploration

The impact of network infrastructure on different groups may vary depending on the needs of the industry, and this difference is particularly evident in the industries in which they are engaged, especially as some specific groups may have a higher demand for comprehensive network application skills. Which occupational groups are mainly facilitated by cyberinfrastructure to cross the competence divide? To further explore this question, the article divides the groups into student group, agricultural work group, non-agricultural work group and entrepreneurship group to explore the heterogeneity of cyberinfrastructure on crossing the competence divide for different groups. The results in Table 8 show that the contribution of cyberinfrastructure to crossing the competence divide is significant at the 1% level of significance for the student group and the non-agricultural work group, significant at the 5% level of significance for the entrepreneurship group, and insignificant for the agricultural work group.

**Table 8:** Occupational heterogeneity.

Variables	Student groups	Agricultural work groups	Non-farm work groups	Entrepreneurial groups
	(1)	(2)	(3)	(4)
Is it a pilot $\times$ Whether to start implementing the policy (with a one-period lag)	0.364*** (0.054)	0.204 (0.039)	0.576*** (0.087)	0.324** (0.066)
Control variables	Yes	Yes	Yes	Yes
Time fixed effects	Yes	Yes	Yes	No
Regional fixed effects	Yes	Yes	Yes	Yes
Observations	2956	8125	3964	3053
${\mathrm{R}}^{2}$	0.128	0.089	0.239	0.187

*, ** and *** indicate significant at the 10%, 5% and 1% levels respectively, with standard errors in brackets.

Possible reasons for this are that, on the one hand, the network infrastructure facilitates the ability of the student population to use online resources for learning, including the use of online classes in schools, etc. The same gives farmers the possibility to master participation in non-farm jobs that require relevant skills and to start their own businesses. On the other hand, these groups also push themselves to cross the competence divide because of their own learning work needs. This is where the importance of network infrastructure to cross the competence divide comes into play. For the groups involved in agricultural work, the ineffectiveness of the current network infrastructure in facilitating their crossing of the competence divide is also evidence of the lack of ability to use digital technology in Chinese agricultural production. To achieve high-quality agricultural development and the transformation of traditional agriculture to modern agriculture it is necessary to address this status quo by applying modern technologies to agricultural production and cultivating new types of farmers. Therefore, how to help the agricultural workforce cross the digital divide is also the next step of the authors' research.

Population aging is a social problem we have to face, the speed of technological progress and equipment upgrade has far exceeded the acceptable range of the elderly groups, the elderly groups are gradually marginalized in the digital process of the whole society, and the digital divide in front of the elderly groups is deepening^23^, and the rural elderly group is even more generally suffering from lower quality level and limited learning ability, which makes the rural elderly group excluded and difficult to enjoy the dividends brought by the digital economy, which is also a difficult problem that must be solved on the road to achieving common prosperity. Can the construction of network infrastructure change this situation, and what is the effect of its impact on different age groups?

Reference to the research experience of NING and LI (2022)^24^, Table 9 takes 30 and 60 years old as age nodes and divides farmers into young, middle-aged and old age groups to explore the effect of the impact of network infrastructure on farmers' ability to cross the digital divide among different age groups. Considering that the older group has little need to learn and work via the Internet, the heterogeneity regarding the capability divide is not analyzed in this section. The results show that network infrastructure in the youth and middle-aged groups facilitates farmers to cross the digital divide at the 1% significance level, while the effect on the older group is not significant. The estimated coefficient of facilitating the middle-aged group to cross the access divide is 2.258, which is much larger than that of the youth group at 0.619. In summary, the effect of network infrastructure to help farmers cross the digital divide is mainly reflected in the middle-aged and youth groups, and the effect on the older group is not significant, and the network infrastructure construction is particularly effective for the middle-aged group to cross the access divide. This is mainly due to the fact that young and middle-aged groups are more capable of accepting new things, and therefore have a stronger advantage in accepting and using the Internet, so the network infrastructure is more obvious to young and middle-aged groups in crossing the digital divide. Similarly, the youth group has a stronger willingness to access the Internet, and therefore the proportion of those who do not rely on digital infrastructure construction to cross the access divide is relatively larger, so the role of digital infrastructure in facilitating farmers to cross the access divide is particularly prominent in the middle-aged group. Through further analysis of the data, it is found that the proportion of the population crossing the access divide and the use divide for the elderly group is close to 0 in 2010, and the proportion of the population crossing the access divide is 4.78% in 2018, while the proportion of the population crossing the use divide is only 0.56%, which is still at a very low level. Therefore, in the era of digital economy, exploring how to help older people cross the digital divide is an issue worth studying. The "Silver Age Crossing the Digital Divide" science popularization initiative launched in 2020 in Zhejiang Province provides free training for the elderly through online courses and offline technology volunteer teaching, which also provides ideas for the authors' later research.

**Table 9:** Age heterogeneity.

Variables	Youth Group		Middle age group		Senior Group	
	Access divide	Use of the divide	Access divide	Use of the divide	Access divide	Use of the divide
Is it a pilot $\times$ Whether to start implementing the policy	0.619*** (0.171)	0.707*** (0.162)	2.258*** (0.351)	0.622*** (0.093)	0.040 (0.078)	0.017 (0.013)
Control variables	Yes	Yes	Yes	Yes	Yes	Yes
Time fixed effects	Yes	Yes	Yes	Yes	Yes	Yes
Regional fixed effects	Yes	Yes	Yes	Yes	Yes	Yes
Observations	3562	3562	9637	9637	4899	4899
${\mathrm{R}}^{2}$	0.212	0.202	0.123	0.128	0.089	0.087

*, ** and *** indicate significant at the 10%, 5% and 1% levels respectively, with standard errors in brackets.

## Conclusions and recommendations

### Conclusions

To achieve the vision of common prosperity, the digital divide that exists in rural areas must be addressed. Network infrastructure plays an important role in raising the income of rural households, narrowing the urban–rural gap, and alleviating relative poverty, but more importantly, it can promote the popularity of the Internet and the development of the digital economy, helping rural households to cross the digital divide. The article uses the 2010–2018 China Family Panel Studies (CFPS) data as a quasi-natural experiment with the help of policy shocks brought by the "broadband countryside" pilot project, and uses the PSM-DID model to estimate the impact of network The impact of infrastructure development on the digital divide is estimated using a PSM-DID model. We further investigate the heterogeneity of network infrastructure on the digital divide by grouping farmers by their age, and summarize the main findings as follows.

(i) Network infrastructure can help farmers cross the digital divide. The "broadband countryside" pilot project has made significant achievements since its launch, and the growth rate of the digital divide in the pilot areas has increased faster than that in the non-pilot areas, achieving a reversal of the crossing rate. Using the "broadband village" strategy as a proxy variable for network infrastructure, we find that network infrastructure can help farmers cross the access and usage divide, but the effect of helping farmers cross the ability divide is not significant. Further analysis found that technical skills training could improve the contribution of network infrastructure to crossing the capability gap.
(ii) Crossing the use divide is the basis for crossing the ability divide. Farmers crossing the capability gap has a significant contribution to the farmers crossing the capability gap in the next period, and farmers need to cross the capability gap as the basis for crossing the use gap, and the average crossing period is about 1.28 periods. Combined with the time costs associated with technical skills training, the response period of farmers crossing the ability gap to policy implementation is longer.
(iii) There is age and occupational heterogeneity in network infrastructure facilitating farmers to cross the digital divide. Network infrastructure facilitates the young and middle-aged groups to cross the access divide and the usage divide, and has the greatest impact on the middle-aged group to cross the access divide, but cannot help the older group to cross the digital divide. As of 2018, the proportion of older groups crossing the access divide and usage divide was only 4.87% and 0.56%. Network infrastructure mainly facilitates the student group, non-agricultural work group and entrepreneurship group to cross the capability divide, and does not have a significant impact on the group involved in agricultural work.

### Recommendations

Based on these findings, the article makes the following recommendations.

(i) Continuously promote the construction of rural network infrastructure. Network infrastructure has a positive role in helping farmers cross the digital divide, but the overall crossing rate is still at a low level. We should continue to promote the construction of rural network infrastructure and increase investment to promote a steady increase in the crossing rate.
(ii) Multiple initiatives to accelerate farmers across the ability gap. It is a long-term process for farmers to cross the capability gap. To accelerate farmers to cross the capability gap, it is necessary not only to meet certain requirements of network infrastructure hardware facilities, but also to improve the overall quality of individual farmers. While promoting the construction of network infrastructure, the government should strengthen the public welfare training of digital technology and network skills, strengthen the construction of digital villages, teach farmers how to apply digital technology skills to agricultural production, non-farm employment and other fields, and make farmers develop the habit of lifelong learning.
(iii) Focus on solving the problem of digital divide among vulnerable groups. The problem of digital divide among rural elderly groups is difficult to be solved through network infrastructure construction, and it is necessary to create electronic products and intelligent services specifically for the elderly groups according to their heterogeneous needs; carry out public information education, teach the elderly groups how to use information technology and improve intelligent skills through media publicity and assigning volunteers, etc. Carry out skills training related to digital applications specifically for agriculture, speed up the transfer of agricultural land to achieve large-scale land management, and encourage farmers to apply digital applications to agricultural production.

## Data Availability

The data has been uploaded in the form of link https://figshare.com/articles/dataset/Digital_divide_data_extraction/24073014.
